# Supplementary material for: Clinical, neuropathological, and biochemical characterization of ALS in a large CHCHD10 R15L family
Source: medRxiv. 2025 Sep 26:2025.09.22.25335938. Preprint. [Version 1] doi: 10.1101/2025.09.22.25335938 (PMC12486031; doi:10.1101/2025.09.22.25335938)

Figure S1

A

Contact corresponding author with requests for data on the extended pedigree.

B

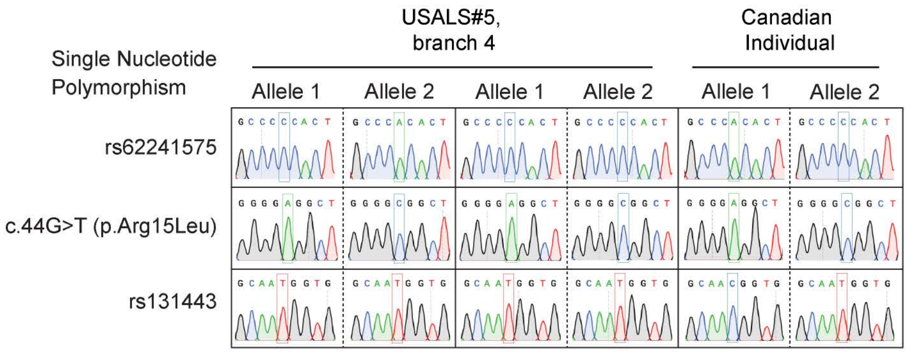

medRxiv preprint doi: https://doi.org/10.1101/2025.09.22.25335938; this version posted September 26, 2025. The copyright holder for this preprint (which was not certified by peer review) is the author/funder, who has granted medRxiv a license to display the preprint in perpetuity. This article is a US Government work. It is not subject to copyright under 17 USC 105 and is also made available for use under a CC0 license.

Figure S2

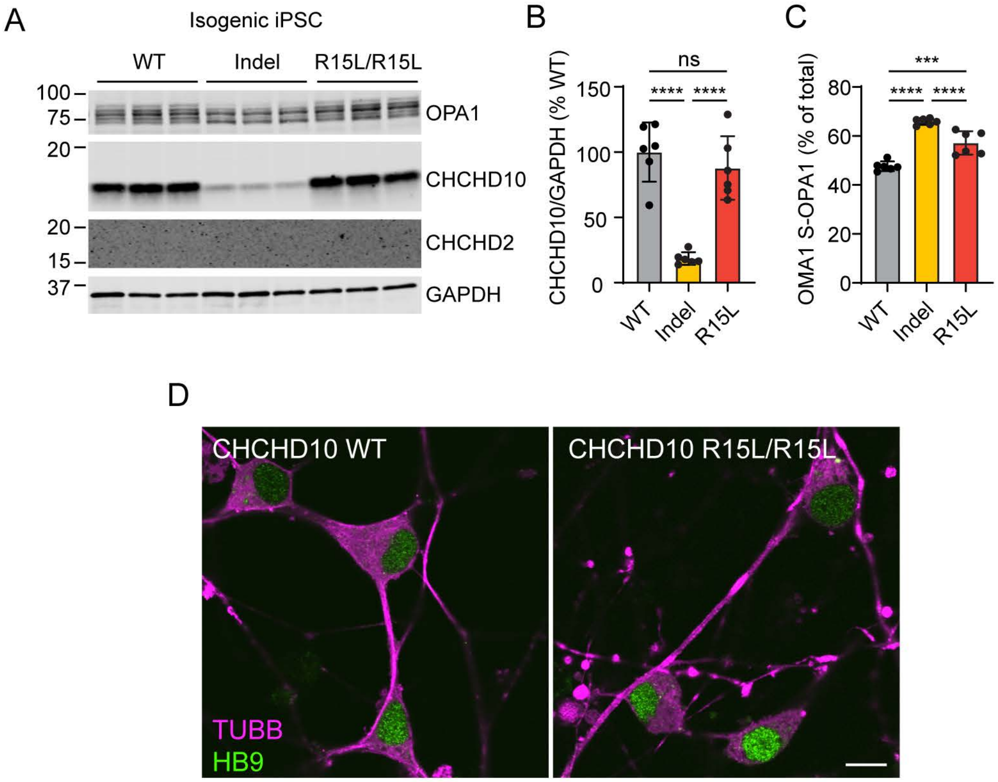

medRxiv preprint doi: https://doi.org/10.1101/2025.09.22.25335938; this version posted September 26, 2025. The copyright holder for this preprint (which was not certified by peer review) is the author/funder, who has granted medRxiv a license to display the preprint in perpetuity. This article is a US Government work. It is not subject to copyright under 17 USC 105 and is also made available for use under a CC0 license.

Figure S3

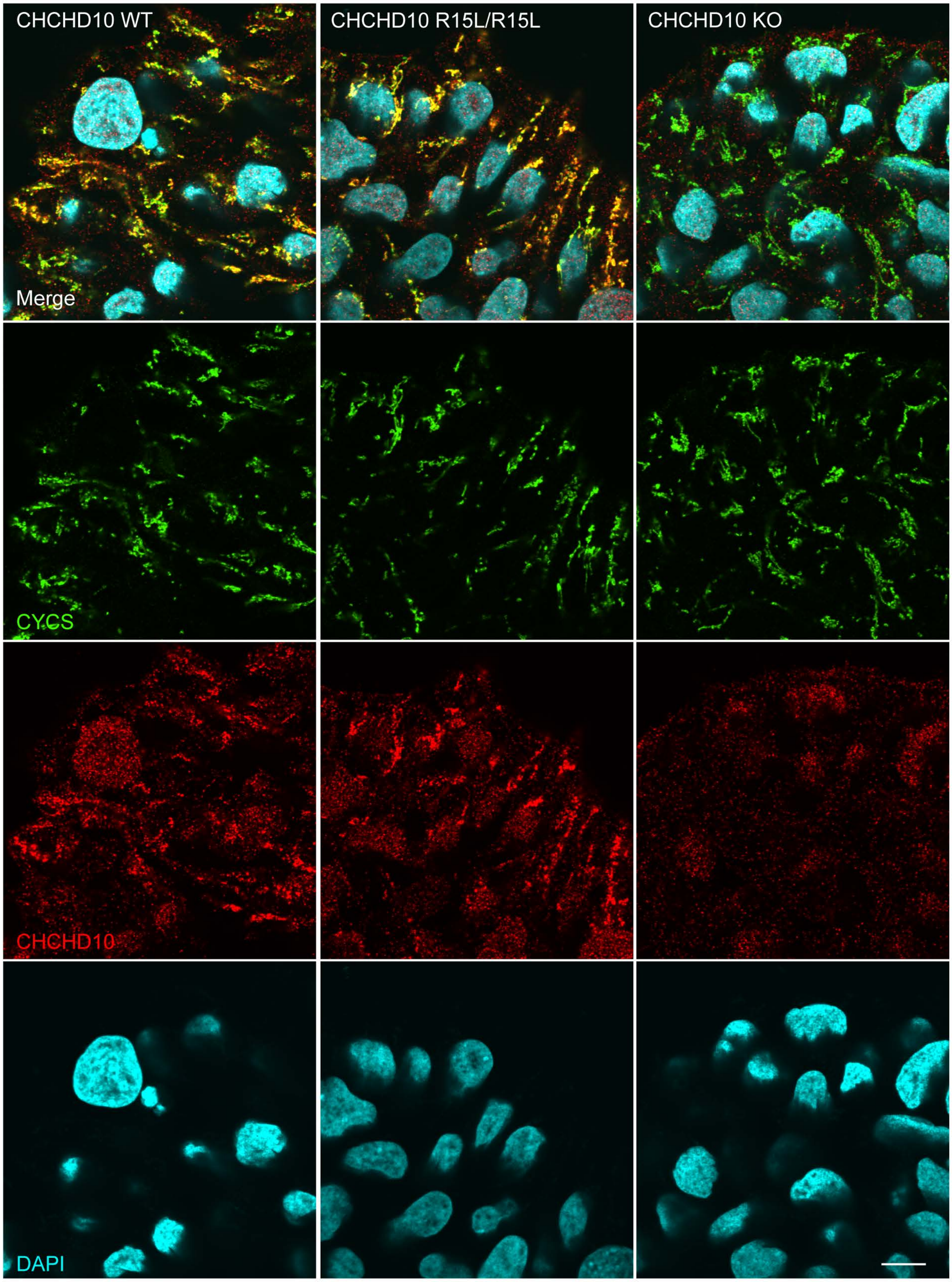

medRxiv preprint doi: https://doi.org/10.1101/2025.09.22.25335938; this version posted September 26, 2025. The copyright holder for this preprint (which was not certified by peer review) is the author/funder, who has granted medRxiv a license to display the preprint in perpetuity. This article is a US Government work. It is not subject to copyright under 17 USC 105 and is also made available for use under a CC0 license.

Figure S4

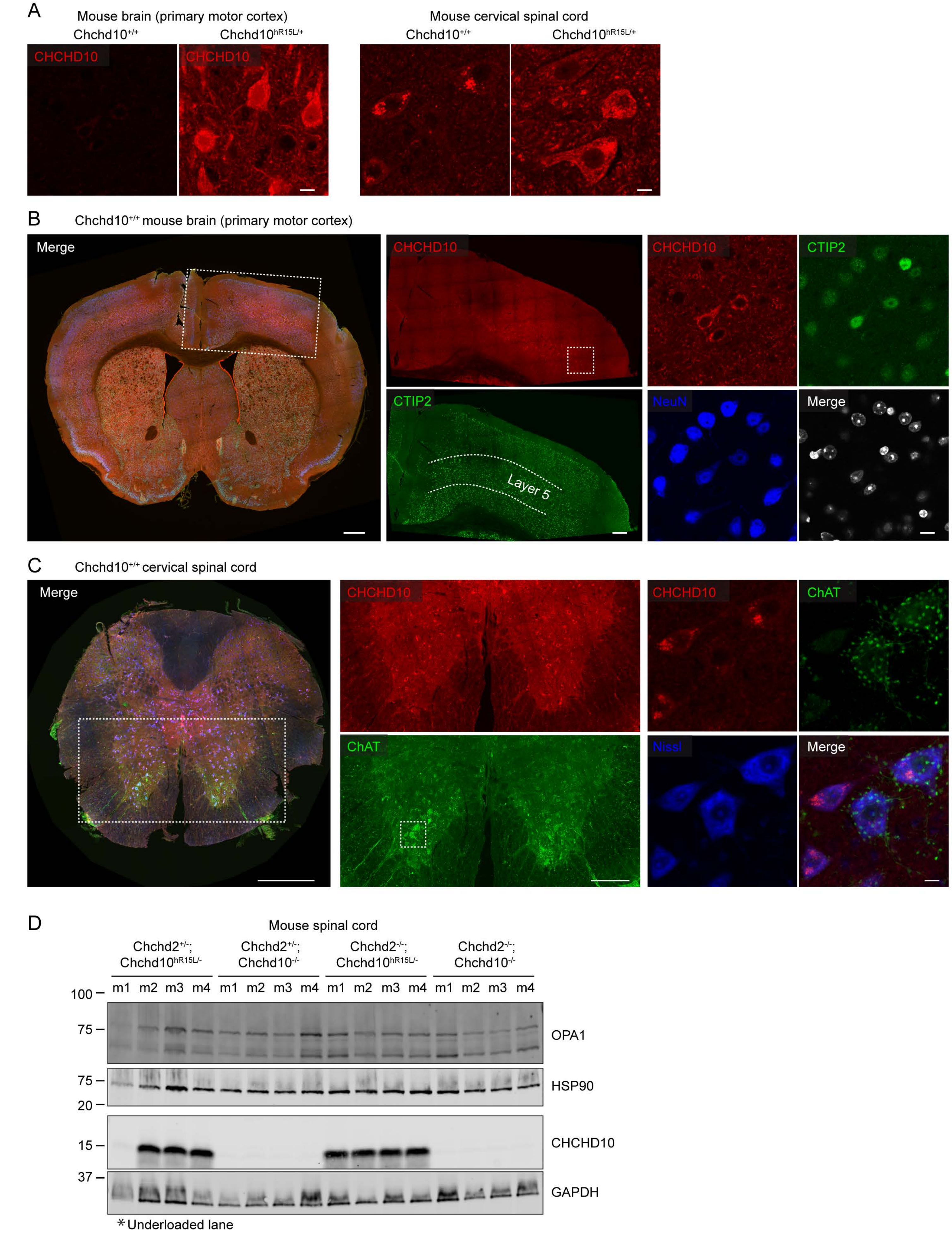

medRxiv preprint doi: https://doi.org/10.1101/2025.09.22.25335938; this version posted September 26, 2025. The copyright holder for this preprint (which was not certified by peer review) is the author/funder, who has granted medRxiv a license to display the preprint in perpetuity.
This article is a US Government work. It is not subject to copyright under 17 USC 105 and is also made available for use under a CC0 license.

Figure S5

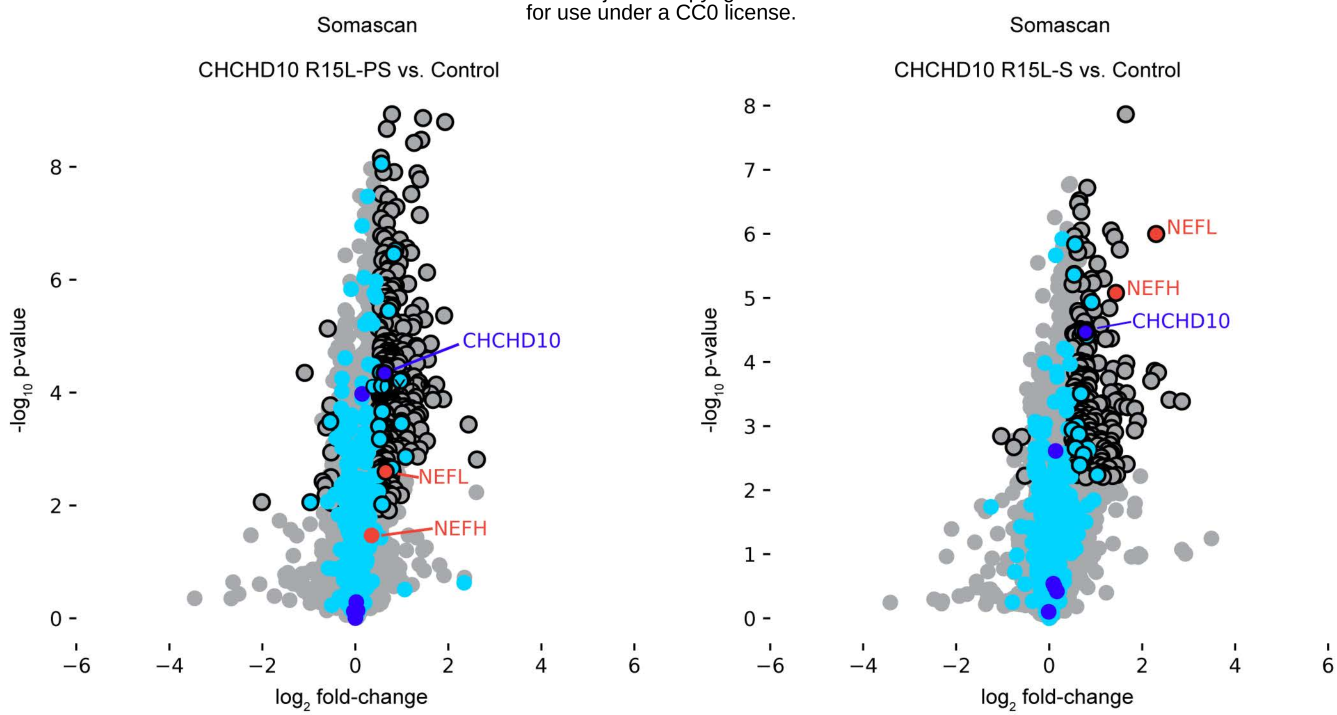

Supplement: 1 — Figure S1. Extended pedigree from CHCHD10 R15L family. (A) Contact corresponding author with requests for data on the extended pedigree. (B) Sanger sequencing of cloned CHCHD10 alleles from indicated family members. Representative sequencing traces are shown across the pathogenic SNP and two SNPs that differed between the haplotype in the USALS#5 family and a previously reported Canadian individual with ALS, who was not known to be related to the USALS#5 family. Figure S2. Comparison of indel and R15L/R15L isogenic iPSC cell lines. (A and B) CHCHD10 protein levels (A and B) and OPA1 cleavage by OMA1 (A and C) were measured by immunoblotting for isogenic WT, CHCHD10 indel, and CHCHD10 R15/R15L iPSC cell lines. OMA1 activation is reflected by the cleavage of L-OPA1 (a and b isoforms) to specific S-OPA1 isoforms (c and e isoforms). (D) iLMNs differentiated as in (Fig. 3H) were immunostained for lower motor neuron markers TUBB (magenta) and HB9 (green). Wells from the same differentiation as (Fig. 3H) were used. In all panels, ns, *, **, ***, ***” correspond to not-significant, p ≤ 0.05, p ≤ 0.01, p ≤ 0.001, and p ≤ 0.0001, respectively. Figure S3. CHCHD10 R15L subcellular localization in iPSC cells. The subcellular localization of CHCHD10 (red) in isogenic parent, R15L/R15L, KO iPSC cells was determined by immunocytochemistry. CHCHD10 co-localized with the mitochondrial marker cytochrome c (CYCS) in both parent and R15L/R15L cells. Some CHCHD10 background staining was present in the nucleus and cytosol in all three lines including the KO. Scale bar = 10 μm. Figure S4. CHCHD10 R15L expression in mouse brain and spinal cord. (A) Comparison of CHCHD10 immunostaining intensity (red) between Chchd10hR15L/+ and Chchd10+/+ brains and spinal cords. Images were windowed the same and the same acquisition parameters were used. The more intense staining in Chchd10hR15L/+ is likely due to higher affinity of the CHCHD10 antibody for human CHCHD10. The images shown here also appear i [file NIHPP2025.09.22.25335938V1-supplement-1.pdf]
